# Some New Traveling Wave Exact Solutions of the (2+1)-Dimensional Boiti-Leon-Pempinelli Equations

**DOI:** 10.1155/2014/743254

**Published:** 2014-02-11

**Authors:** Jian-ming Qi, Fu Zhang, Wen-jun Yuan, Zi-feng Huang

**Affiliations:** ^1^Department of Mathematics and Physics, Shanghai Dianji University, Shanghai 201306, China; ^2^School of Mathematics and Information Science, Guangzhou University, Guangzhou 510006, China

## Abstract

We employ the complex method to obtain all meromorphic exact solutions of complex (2+1)-dimensional Boiti-Leon-Pempinelli equations (BLP system of equations). The idea introduced in this paper can be applied to other nonlinear evolution equations. Our results show that all rational and simply periodic traveling wave exact solutions of the equations (BLP) are solitary wave solutions, the complex method is simpler than other methods, and there exist some rational solutions *u*_*r*,2_ *(z)* and simply periodic solutions *u*_*s*,2–6_(*z*) which are not only new but also not degenerated successively by the elliptic function solutions. We believe that this method should play an important role for finding exact solutions in the mathematical physics. For these new traveling wave solutions, we give some computer simulations to illustrate our main results.

## 1. Introduction

Boiti et al. [1] introduced the Boiti-Leon-Pempinelli equations (BLP system of equations)

(1) $$\begin{array}{c} u_{ty}={\left(u^{2}-u_{x}\right)}_{xy}+2v_{xxx}, \end{array}$$

(2) $$\begin{array}{c} v_{t}=v_{xx}+2uv_{x}. \end{array}$$

A considerable research work has been invested in [2–5] to study the BLP system (1) and (2). The integrability of this system was studied in [1] by using the Sine-Gordon and the Sinh-Gordon equations. Other works have been conducted by using other methods such as Jacobi elliptic methods and balance methods [3–5].

For finding exact solutions of the BLP system, many authors applied the tanh-coth method and the Exp-function method to derive them [6–17] and [8–22], respectively.

In 2010, Wazwaz and Mehanna [17] used the tanh-coth method and Exp-function method to the BLP equations to derive many new varieties of travelling wave solutions with distinct physical structures. Substituting the traveling wave transformation

(3) $$\begin{array}{c} u\left(x,y,t\right)=u\left(z\right),\;\;v\left(x,y,t\right)=v\left(z\right),\\ z=\mu \left(x+y-ct\right) \end{array}$$

into (1) and (2), one carries out the system of nonlinear ordinary differential equations as follows:

(4) $$\begin{array}{c} -cu^{\prime \prime }={\left(u^{2}\right)}^{\prime \prime }-\mu u^{\prime \prime \prime }+2\mu v^{\prime \prime \prime }, \end{array}$$

(5) $$\begin{array}{c} -cv^{\prime }=\mu v^{\prime \prime }+2uv^{\prime }, \end{array}$$

where *c* is the wave velocity and *μ* is a nonzero constant (see [17, 23]).

Afterwards we integrated (1) twice with respect to *z* and considered the constants of integration to be zero and obtained

(6) $$\begin{array}{c} v^{\prime }=\frac{1}{2}u^{\prime }-\frac{u^{2}+cu}{2\mu }. \end{array}$$

Furthermore substituting (6) into (5) yields

(7) $$\begin{array}{c} \mu^{2}u^{\prime \prime }-2u^{3}-3cu^{2}-c^{2}u=0. \end{array}$$

In 2011, Kudryashov [23] got the general solutions of (7) via Jacobi elliptic functions and analyzed the application of the tanh-coth method for finding exact solutions of (7) and showed that all the solutions which are presented by Wazwaz and Mehanna can be reduced to a single one and so on.

In this paper, we employ the complex method which was introduced by Yuan et al. [24–26] to obtain the general solutions and some new solutions of (7). In order to state our results, we need some concepts and notations.

A meromorphic function *w*(*z*) means that *w*(*z*) is holomorphic in the complex plane *ℂ* except for poles. *℘*(*z*; *g*_2_, *g*_3_) is the Weierstrass elliptic function with invariants *g*_2_ and *g*_3_. We say that a meromorphic function *f* belongs to the class *W* if *f* is an elliptic function, or a rational function of *e* ^*αz*^, *α* ∈ *ℂ*, or a rational function of *z*.

Our main result is the following theorem.

**Theorem**

All meromorphic solutions *u* of (7) belong to the class *W*. Furthermore, (7) has the following three forms of solutions.

(I) The elliptic general solutions

(8) wd(z)=±μ2 ×(((−℘+A)(4℘A2+4℘2A          +2℘′B−℘g2−Ag2))   ×(((12A2−g2)℘+4A3−3Ag2)℘′      +4B℘3+12AB℘2−3Bg2℘−ABg2)−1),

where *g*_3_ = 0, *B* ^2^ = 4*A* ^3^ − *g*_2_ *A*, and *g*_2_ and *A* are arbitrary constants.

(II) The simply periodic solutions, where *ξ* = *e* ^*αz*^ are obtained, for *z*_0_ ∈ *ℂ*,

(9) $$\begin{array}{c} u_{s,1}\left(z\right)=\frac{c}{e^{\alpha z}-1}=\frac{c}{2}\left(\coth\frac{\alpha }{2}\left(z-z_{0}\right)-1\right), \end{array}$$

where *μ* = ±*c*/*α*;

(10) $$\begin{array}{c} u_{s,2}\left(z\right)=-\frac{c}{2}\left(1+\coth\frac{\alpha }{2}\left(z-z_{0}\right)\right), \end{array}$$

where *μ* = ±*c*/*α*;

(11) $$\begin{array}{c} u_{s,3}\left(z\right)=c\coth\alpha \left(z-z_{0}\right)-c, \end{array}$$

where *μ* = ±*c*/2*α*;

(12) $$\begin{array}{c} u_{s,4}\left(z\right)=-c\coth\alpha \left(z-z_{0}\right), \end{array}$$

where *μ* = ±*c*/2*α*;

(13) $$\begin{array}{c} u_{s,5}\left(z\right)=\frac{\sqrt{2}ci}{2\text{Sinh}\alpha \left(z-z_{0}\right)}-\frac{c}{2}, \end{array}$$

where $\mu =\pm \sqrt{2}ci/2\alpha$;

(14) $$\begin{array}{c} u_{s,6}\left(z\right)=-\frac{\sqrt{2}ci}{2\text{Sinh}\alpha \left(z-z_{0}\right)}-\frac{c}{2}, \end{array}$$

where $\mu =\pm \sqrt{2}ci/2\alpha$.

(III) All rational function solutions are of the following two distinct forms. For any *z*_0_ ∈ *ℂ*,

(15) $$\begin{array}{c} u_{r,1}\left(z\right)=\pm \frac{\mu }{z-z_{0}},\\ u_{r,2}\left(z\right)=\pm \frac{\mu }{z-z_{0}}\pm \frac{\mu }{z-z_{0}-z_{1}}, \end{array}$$

where *z*_0_ ∈ *ℂ*, *z*_1_ ≠ 0, *c* = 0.

## 2. Preliminary Lemmas and the Complex Method

In order to give complex method and the proof of Theorem 1, we need some notations and results.

Set *m* ∈ *ℕ* : = {1,2, 3,…}, *r*_*j*_ ∈ *ℕ*_0_ = *ℕ* ∪ {0}, *r* = (*r*_0_, *r*_1_,…, *r*_*m*_), *j* = 0,1,…, *m*. We define a differential monomial denoted by

(16) Mr[w](z):=[w(z)]r0[w′(z)]r1 ×[w′′(z)]r2⋯[w(m)(z)]rm.

*p*(*r*): = *r*_0_ + 2*r*_1_ + ⋯+(*m* + 1)*r*_*m*_ and deg⁡(*M*) are called the weight and degree of *M*_*r*_[*w*], respectively.

A differential polynomial *P*(*w*, *w*′,…, *w* ^(*m*)^) is defined as follows:

(17) $$\begin{array}{c} P\left(w,w^{\prime },\ldots ,w^{\left(m\right)}\right):=\sum_{r\in I}a_{r}M_{r}\left[w\right], \end{array}$$

where *a*_*r*_ are constants and *I* is a finite index set. The total weight and degree of *P*(*w*, *w*′,…, *w* ^(*m*)^) are defined by *W*(*P*): = max⁡_*r*∈*I*_{*p*(*r*)} and deg⁡(*P*): = max⁡_*r*∈*I*_{deg⁡(*M*_*r*_)}, respectively.

We will consider the following complex ordinary differential equations:

(18) $$\begin{array}{c} P\left(w,w^{\prime },\ldots ,w^{\left(m\right)}\right)=bw^{n}+c, \end{array}$$

where *b* ≠ 0, *c* are constants, *n* ∈ *ℕ*.

Let *p*, *q* ∈ *ℕ*. Suppose that (18) has a meromorphic solution *w* with at least one pole; we say that (18) satisfies weak 〈*p*, *q*〉 condition if, substituting Laurent series

(19) $$\begin{array}{c} w\left(z\right)=\sum_{k=-q}^{\infty }c_{k}z^{k},\;q>0,\;c_{-q}\neq 0 \end{array}$$

into (18), we can determine *p* distinct Laurent singular parts below

(20) $$\begin{array}{c} \sum_{k=-q}^{-1}c_{k}z^{k}. \end{array}$$

**Lemma 2**

Let *p*, *l*, *m*, *n* ∈ *ℕ*, deg⁡*P*(*w*, *w* ^(*m*)^) < *n*. Suppose that an *m* order Briot-Bouquet equation

(21) $$\begin{array}{c} P\left(w^{\left(m\right)},w\right)=bw^{n}+c \end{array}$$

satisfies weak 〈*p*, *q*〉 condition, then whose all meromorphic solutions *w* belong to the class *W*. If for some values of parameters such solution *w* exists, then other meromorphic solutions form a one parametric family *w*(*z* − *z*_0_), *z*_0_ ∈ *ℂ*. Furthermore each elliptic solution with pole at *z* = 0 can be written as

(22) w(z)=∑i=1l−1 ∑j=2qi(−1)jc−ij(j−1)!dj−2dzj−2‍     ×(14[℘′(z)+Bi℘(z)−Ai]2−℘(z)) +∑i=1l−1c−i12℘′(z)+Bi℘(z)−Ai +∑j=2ql(−1)jc−lj(j−1)!dj−2dzj−2℘(z)+c0,

where *c*_−*ij*_ are given by (19), *B*_*i*_^2^ = 4*A*_*i*_^3^ − *g*_2_ *A*_*i*_ − *g*_3_, and ∑_*i*=1_^*l*^ *c*_−*i*1_ = 0.

Each rational function solution *w* : = *R*(*z*) is of the form

(23) $$\begin{array}{c} R\left(z\right)=\sum_{i=1}^{l}\;\sum_{j=1}^{q}\frac{c_{ij}}{{\left(z-z_{i}\right)}^{j}}+c_{0}, \end{array}$$

with *l*(≤*p*) distinct poles of multiplicity *q*.

Each simply periodic solution is a rational function *R*(*ξ*) of *ξ* = *e* ^*αz*^(*α* ∈ *ℂ*). *R*(*ξ*) has *l*(≤*p*) distinct poles of multiplicity *q* and is of the form

(24) $$\begin{array}{c} R\left(\xi \right)=\sum_{i=1}^{l}\;\sum_{j=1}^{q}\frac{c_{ij}}{{\left(\xi -\xi_{i}\right)}^{j}}+c_{0}. \end{array}$$

In order to give the representations of elliptic solutions, we need some notations and results concerning elliptic function [27].

Let *ω*_1_, *ω*_2_ be two given complex numbers such that *Im*⁡*ω*_1_/*ω*_2_ > 0, *L* = *L*[2*ω*_1_, 2*ω*_2_] be discrete subset *L*[2*ω*_1_, 2*ω*_2_] = {*ω* | *ω* = 2*nω*_1_ + 2*mω*_2_, *n*, *m* ∈ *ℤ*}, which is isomorphic to *ℤ* × *ℤ*. The discriminant Δ = Δ(*c*_1_, *c*_2_): = *c*_1_^3^ − 27*c*_2_^2^ and

(25) $$\begin{array}{c} s_{n}=s_{n}\left(L\right):=\sum_{\omega \in L\setminus \left\{0\right\}}\frac{1}{\omega^{n}}. \end{array}$$

Weierstrass elliptic function *℘*(*z*): = *℘*(*z*, *g*_2_, *g*_3_) is a meromorphic function with double periods 2*ω*_1_, 2*ω*_2_ and satisfying the equation

(26) $$\begin{array}{c} {\left({℘}^{\prime }\left(z\right)\right)}^{2}=4℘{\left(z\right)}^{3}-g_{2}℘\left(z\right)-g_{3}, \end{array}$$

where *g*_2_ = 60*s*_4_, *g*_3_ = 140*s*_6_ and Δ(*g*_2_, *g*_3_) ≠ 0.

If we change (24) to the form

(27) $$\begin{array}{c} {\left({℘}^{\prime }\left(z\right)\right)}^{2}=4\left(℘\left(z\right)-e_{1}\right)\left(℘\left(z\right)-e_{2}\right)\left(℘\left(z\right)-e_{2}\right), \end{array}$$

we have *e*_1_ = *℘*(*ω*_1_), *e*_2_ = *℘*(*ω*_2_), and  *e*_3_ = *℘*(*ω*_1_ + *ω*_2_).

Inversely, given two complex numbers *g*_2_ and *g*_3_ such that Δ(*g*_2_, *g*_3_) ≠ 0, then there exists double periods 2*ω*_1_, 2*ω*_2_ Weierstrass elliptic function *℘*(*z*) such that the above results hold.

**Lemma (see [27, 28])**

Weierstrass elliptic functions *℘*(*z*): = *℘*(*z*, *g*_2_, *g*_3_) have two successive degeneracies and addition formula.

(I) Degeneracy to simply periodic functions (i.e., rational functions of one exponential *e* ^*kz*^) according to

(28) $$\begin{array}{c} ℘\left(z,3d^{2},-d^{3}\right)=2d-\frac{3d}{2}\coth^{2}\sqrt{\frac{3d}{2}}z, \end{array}$$

if one root *e*_*j*_ is double (Δ(*g*_2_, *g*_3_) = 0).

(II) Degeneracy to rational functions of *z* according to

(29) $$\begin{array}{c} ℘\left(z,0,0\right)=\frac{1}{z^{2}} \end{array}$$

if one root *e*_*j*_ is triple (*g*_2_ = *g*_3_ = 0).

(III) Addition formula

(30) ℘(z−z0)=−℘(z)−℘(z0) +14[℘′(z)+℘′(z0)℘(z)−℘(z0)]2.

By the above lemma and results, we can give a new method below, say *complex method*, to find exact solutions of some PDEs.

**Step**

Substituting the transform *T* : *u*(*x*, *t*) → *w*(*z*), (*x*, *t*) → *z* into a given PDE gives a nonlinear ordinary differential equation (18) or (21).

**Step**

Substitute (19) into (18) or (21) to determine that weak 〈*p*, *q*〉 condition holds.

**Step 3**

By determinant relation (22)–(24) we find the elliptic, rational, and simply periodic solutions *u*(*z*) of (18) or (21) with pole at *z* = 0, respectively.

**Step 4**

By Lemmas 2 and 3 we obtain all the meromorphic solutions *w*(*z* − *z*_0_).

**Step 5**

Substituting the inverse transform *T* ^−1^ into these meromorphic solutions *w*(*z* − *z*_0_), then we get all exact solutions *u*(*x*, *t*) of the original given PDE.

## 3. Proof of Theorem 1

Substituting (19) into (7) we have *q* = 1. *p* = 2, *c*_−1_ = ±*μ*. Hence, (7) satisfies weak 〈2,1〉 condition and is a 2nd order Briot-Bouquet differential equation. Obviously, (7) satisfies the dominant condition. So, by Lemma 2, we know that all meromorphic solutions of (7) belong to *W*. Now we will give the forms of all meromorphic solutions of (7).

By (22), we infer the indeterminant rational solutions of (7) with pole at *z* = 0 that

(31) $$\begin{array}{c} u_{r}\left(z\right)=\frac{c_{11}}{z}+\frac{c_{12}}{z-z_{1}}+c_{10}. \end{array}$$

Substituting *u*_*r*_(*z*) into (7), we get two distinct forms. One of them is

(32) $$\begin{array}{c} u_{r0,1}\left(z\right)=\pm \frac{\mu }{z}, \end{array}$$

where *c* = 0. The other is

(33) $$\begin{array}{c} u_{r0,2}\left(z\right)=\pm \frac{\mu }{z}\pm \frac{\mu }{z-z_{1}}, \end{array}$$

where *c* = 0.

Thus all rational solutions of (7) are

(34) $$\begin{array}{c} u_{r,1}\left(z\right)=\pm \frac{\mu }{z-z_{0}},\\ u_{r,2}\left(z\right)=\pm \frac{\mu }{z-z_{0}}\pm \frac{\mu }{z-z_{0}-z_{1}}, \end{array}$$

where *z*_0_ ∈ *ℂ*, *z*_1_ ≠ 0, *c* = 0.

In order to have simply periodic solutions, set *ξ* = exp⁡(*αz*), put *u* = *u*(*ξ*) into (7), and then

(35) $$\begin{array}{c} \mu^{2}\alpha^{2}\left(\xi^{2}u^{\prime \prime }+\xi u^{\prime }\right)-2u^{3}-3cu^{2}-c^{2}u=0. \end{array}$$

Substituting

(36) $$\begin{array}{c} u_{2}\left(\xi \right)=\frac{c_{2}}{\xi -1}+\frac{c_{1}}{\xi -\xi_{1}}+c_{0} \end{array}$$

into (7), we obtain the indeterminant simply periodic solutions of (35) with pole at *ξ* = 1 that

(37) $$\begin{array}{c} u_{s1,1}\left(\xi \right)=\frac{c}{\xi -1}, \end{array}$$

where *μ* = ±*c*/*α*;

(38) $$\begin{array}{c} u_{s1,2}\left(\xi \right)=-\frac{c}{\xi -1}-c, \end{array}$$

where *μ* = ±*c*/*α*;

(39) $$\begin{array}{c} u_{s1,3}\left(\xi \right)=\frac{c}{2\left(\xi -1\right)}+\frac{-c}{2\left(\xi +1\right)}=c\frac{\xi +\xi^{-1}}{\xi -\xi^{-1}}-c, \end{array}$$

where *μ* = ±*c*/2*α*;

(40) $$\begin{array}{c} u_{s1,4}\left(\xi \right)=\frac{-c}{2\left(\xi -1\right)}+\frac{c}{2\left(\xi +1\right)}-c=-c\frac{\xi +\xi^{-1}}{\xi -\xi^{-1}}, \end{array}$$

where *μ* = ±*c*/2*α*;

(41) $$\begin{array}{c} u_{s1,5}\left(\xi \right)=\frac{\sqrt{2}ci}{2\left(\xi -1\right)}+\frac{\sqrt{2}ci}{2\left(\xi +1\right)}-\frac{c}{2}=\frac{\sqrt{2}ci}{\xi -\xi^{-1}}-\frac{c}{2}, \end{array}$$

where $\mu =\pm \sqrt{2}ci/2\alpha$;

(42) $$\begin{array}{c} u_{s1,6}\left(\xi \right)=-\frac{\sqrt{2}ci}{2\left(\xi -1\right)}-\frac{\sqrt{2}ci}{2\left(\xi +1\right)}-\frac{c}{2}=-\frac{\sqrt{2}ci}{\xi -\xi^{-1}}-\frac{c}{2}, \end{array}$$

where $\mu =\pm \sqrt{2}ci/2\alpha$.

Substitute *ξ* = *e* ^*αz*^ into the above six relations, and then we get all simply periodic solutions of (7) with pole at *z* = 0

(43) $$\begin{array}{c} u_{s0,1}\left(z\right)=\frac{c}{2}\left(\coth\frac{\alpha }{2}z-1\right), \end{array}$$

where *μ* = ±*c*/*α*;

(44) $$\begin{array}{c} u_{s0,2}\left(z\right)=-\frac{c}{2}\left(1+\coth\frac{\alpha }{2}z\right), \end{array}$$

where *μ* = ±*c*/*α*;

(45) $$\begin{array}{c} u_{s0,3}\left(z\right)=c\coth\alpha z-c, \end{array}$$

where *μ* = ±*c*/2*α*;

(46) $$\begin{array}{c} u_{s0,4}\left(z\right)=-c\coth\alpha z, \end{array}$$

where *μ* = ±*c*/2*α*;

(47) $$\begin{array}{c} u_{s0,5}\left(z\right)=\frac{\sqrt{2}ci}{2\text{Sinh}\alpha z}-\frac{c}{2}, \end{array}$$

where $\mu =\pm \sqrt{2}ci/2\alpha$;

(48) $$\begin{array}{c} u_{s0,6}\left(z\right)=-\frac{\sqrt{2}ci}{2\text{Sinh}\alpha z}-\frac{c}{2}, \end{array}$$

where $\mu =\pm \sqrt{2}ci/2\alpha$.

So all simply periodic solutions of (7) are obtained, for *z*_0_ ∈ *ℂ*, by

(49) $$\begin{array}{c} u_{s,1}\left(z\right)=\frac{c}{2}\left(\coth\frac{\alpha }{2}\left(z-z_{0}\right)-1\right), \end{array}$$

where *μ* = ±*c*/*α*;

(50) $$\begin{array}{c} u_{s,2}\left(z\right)=-\frac{c}{2}\left(1+\coth\frac{\alpha }{2}\left(z-z_{0}\right)\right), \end{array}$$

where *μ* = ±*c*/*α*;

(51) $$\begin{array}{c} u_{s,3}\left(z\right)=c\coth\alpha \left(z-z_{0}\right)-c, \end{array}$$

where *μ* = ±*c*/2*α*;

(52) $$\begin{array}{c} u_{s,4}\left(z\right)=-c\coth\alpha \left(z-z_{0}\right), \end{array}$$

where *μ* = ±*c*/2*α*;

(53) $$\begin{array}{c} u_{s,5}\left(z\right)=\frac{\sqrt{2}ci}{2\sinh\alpha \left(z-z_{0}\right)}-\frac{c}{2}, \end{array}$$

where $\mu =\pm \sqrt{2}ci/2\alpha$;

(54) $$\begin{array}{c} u_{s,6}\left(z\right)=-\frac{\sqrt{2}ci}{2\text{Sinh}\alpha \left(z-z_{0}\right)}-\frac{c}{2}, \end{array}$$

where $\mu =\pm \sqrt{2}ci/2\alpha$.

From (21) in Lemma 2, we have indeterminant relations of elliptic solutions of (7) with pole at *z* = 0

(55) $$\begin{array}{c} u_{d0}\left(z\right)=\frac{c_{-1}}{2}\frac{{℘}^{\prime }\left(z\right)+F}{℘\left(z\right)-E}+c_{30}, \end{array}$$

where *F* ^2^ = 4*E* ^3^ − *g*_2_ *E* − *g*_3_. Applying conclusion II of Lemma 2 to *u*_*d*0_(*z*) and noting the results of rational solutions obtained above, we deduce that *c*_30_ = 0, *E* = *F* = 0, and *g*_3_ = 0. Then we get that

(56) $$\begin{array}{c} u_{d0}\left(z\right)=\pm \frac{\mu }{2}\frac{{℘}^{\prime }\left(z\right)}{℘\left(z\right)}. \end{array}$$

Therefore, all elliptic function solutions of (7)

(57) $$\begin{array}{c} u_{d0}\left(z\right)=\pm \frac{\mu }{2}\frac{{℘}^{\prime }\left(z-z_{0}\right)}{℘\left(z-z_{0}\right)}. \end{array}$$

Here *z*_0_ ∈ *ℂ*, *g*_3_ = 0. Making use of the addition of Lemma 3, we rewrite it to the form

(58) wd(z)=±μ2  ×(((−℘+A)(4℘A2+4℘2A         +2℘′B−℘g2−Ag2))  ×(((12A2−g2)℘+4A3−3Ag2)℘′     +4B℘3+12AB℘2−3Bg2℘−ABg2)−1).

Here *g*_3_ = 0,  *B* ^2^ = 4*A* ^3^ − *g*_2_ *A*,  *g*_2_, and *A* are arbitrary constants.

This completes the proof of Theorem 1.

## 4. Computer Simulations for New Solutions

**Figure 1:**
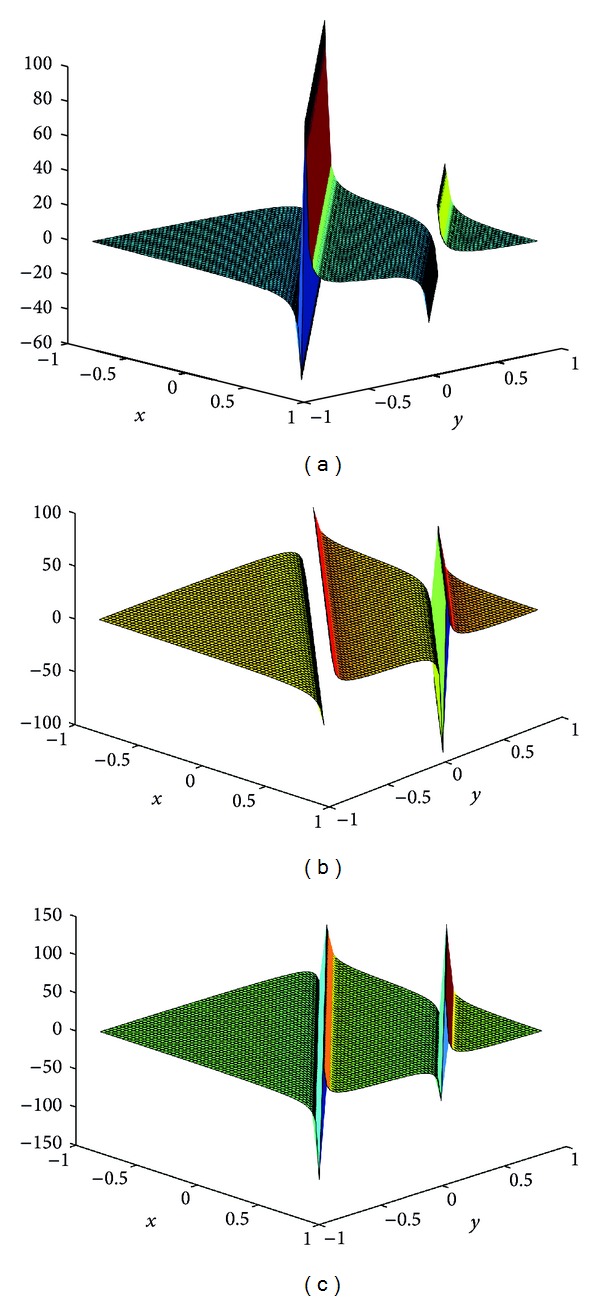
The solution of BLP corresponding to *u*_*r*,2_: take *c* = 1, *μ* = 1, *z*_0_ = 0, *z*_1_ = 1; (a) *t* = −1/100, (b) *t* = 0, and (c) *t* = 1/100.

**Figure 2:**
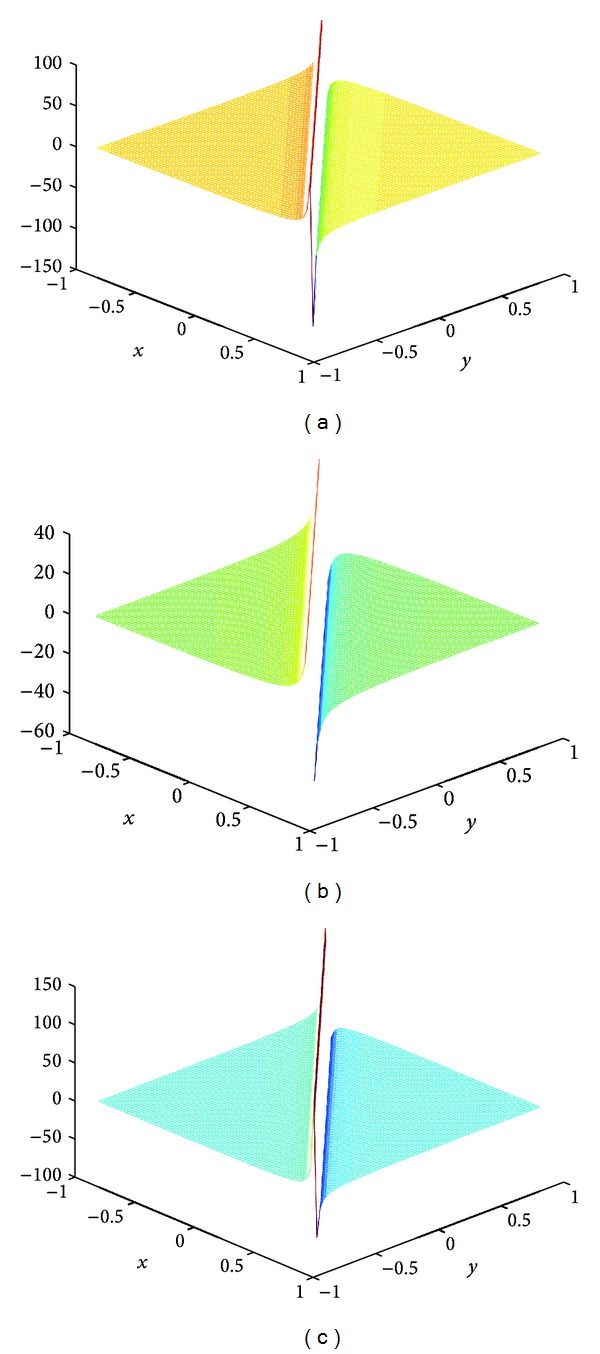
The solution of BLP corresponding to *u*_*s*,2_: take *c* = 1, *α* = 1, *z*_0_ = 0; (a) *t* = −1/115, (b) *t* = 0, and (c) *t* = 1/115.

**Figure 3:**
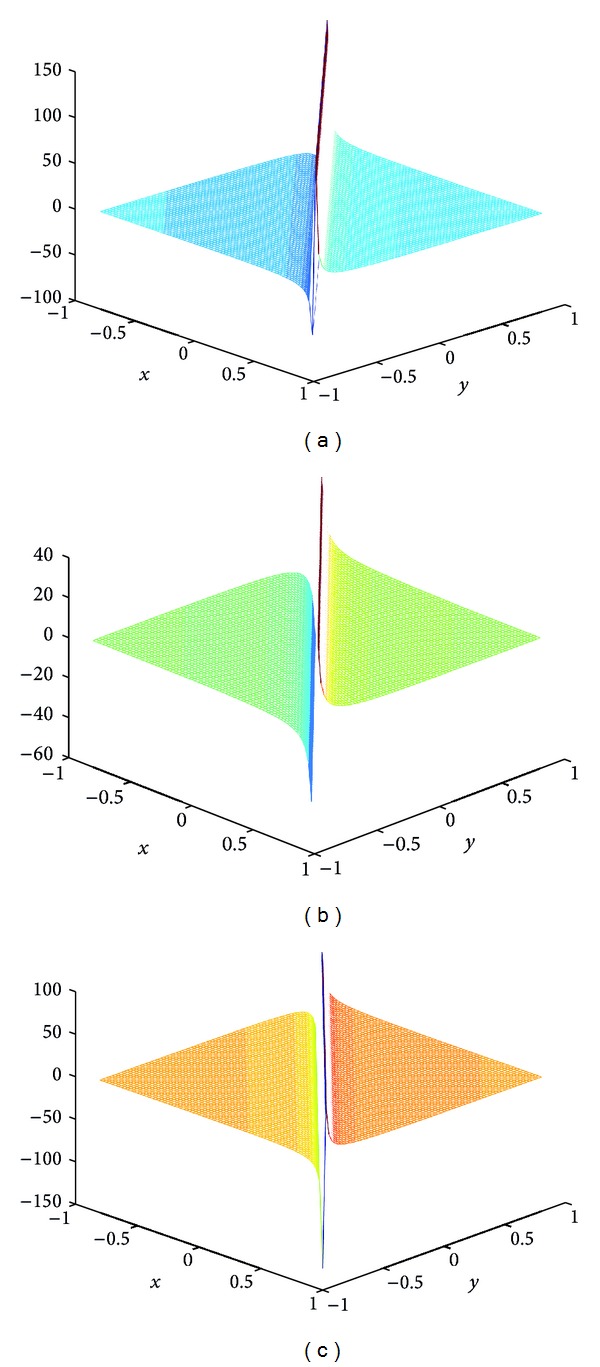
The solution of BLP corresponding to *u*_*s*,3_: take *c* = 1, *α* = 1, *z*_0_ = 0; (a) *t* = −1/132, (b) *t* = 0, and (c) *t* = 1/132.

**Figure 4:**
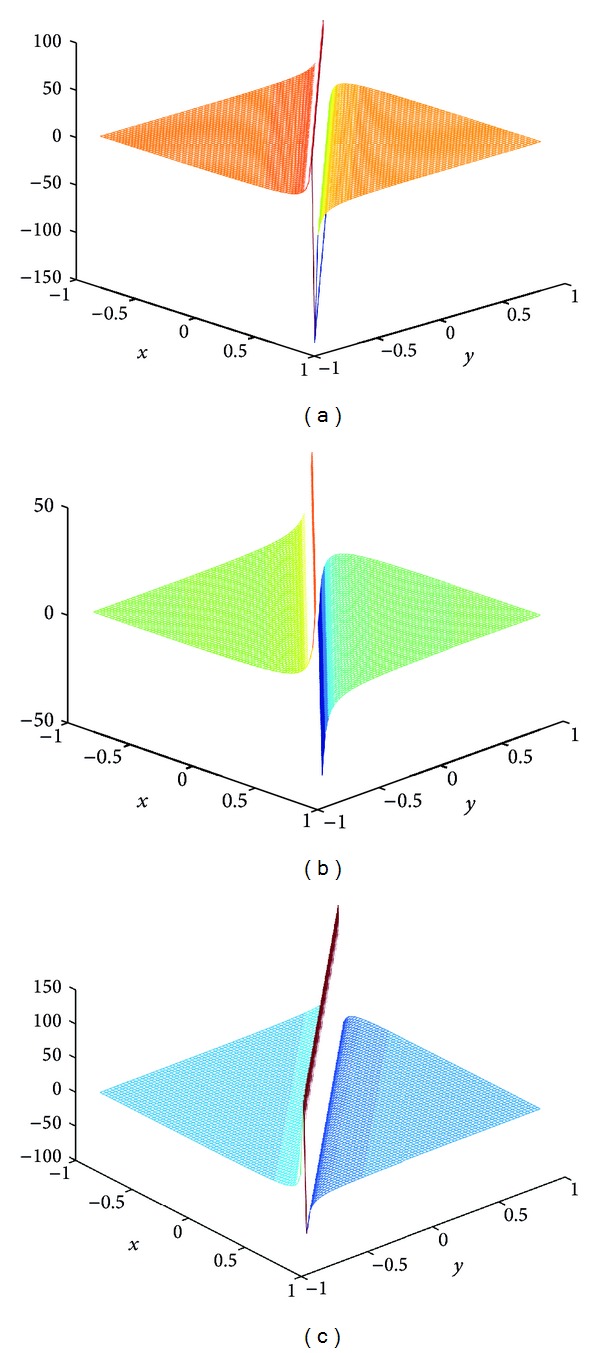
The solution of BLP corresponding to *u*_*s*,4_: take *c* = 1, *α* = 1, *z*_0_ = 0; (a) *t* = −1/144, (b) *t* = 0, and (c) *t* = 1/144.

**Figure 5:**
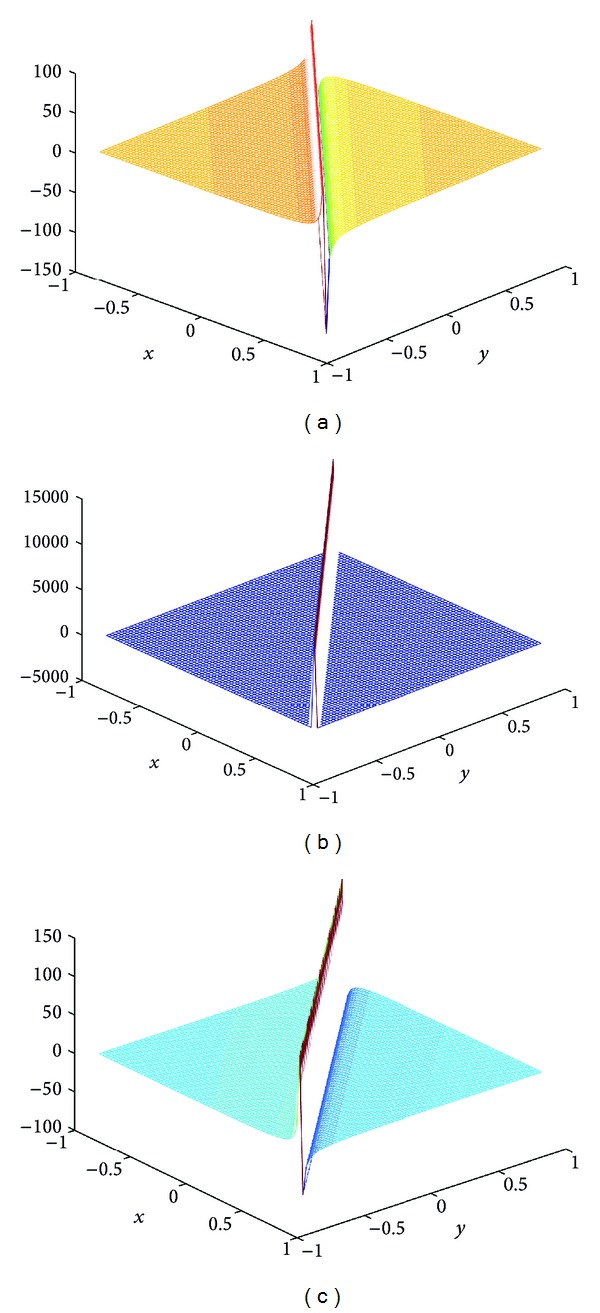
The solution of BLP corresponding to *u*_*s*,5_: take *c* = 1, *α* = 1, *z*_0_ = 0; (a) *t* = −1/125, (b) *t* = 1/10000, and (c) *t* = 1/125.

**Figure 6:**
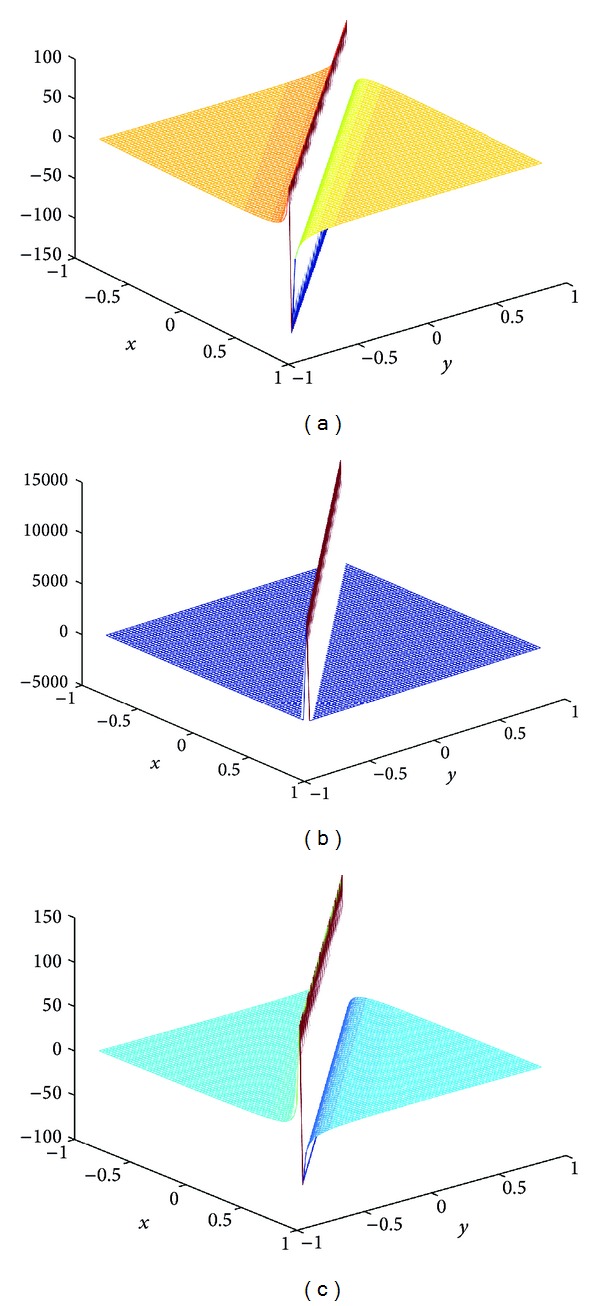
The solution of BLP corresponding to *u*_*s*,6_: take *c* = 1, *α* = 1, *z*_0_ = 0; (a) *t* = −1/123, (b) *t* = 1/10000, and (c) *t* = 1/123.

In this section, we give some computer simulations to illustrate our main results. Here we take the new rational solutions *u*_*r*,2_(*z*) and simply periodic solutions *u*_*s*,2–6_(*z*) to further analyze their properties by Figures 1, 2, 3, 4, 5, and 6.

## 5. Conclusions

Complex method is a very important tool in finding the exact solutions of nonlinear evolution equations, and the (2+1)-dimensional Boiti-Leon-Pempinelli equation is classic and simplest case of the nonlinear reaction-diffusion equation. In this paper, we employ the complex method to obtain the general meromorphic solutions of the (2+1)-dimensional Boiti-Leon-Pempinelli equation, which improves the corresponding result obtained by Kudryashov [23] and Wazwaz and Mehanna [17]. Our results show that all rational and simply periodic traveling wave exact solutions of the equations (BLP) are solitary wave solutions, the complex method is simpler than other methods, and there exist some rational solutions *u*_*r*,2_(*z*) and simply periodic solutions *u*_*s*,2–6_(*z*) which are not only new but also not degenerated successively by the elliptic function solutions. We believe that this method should play an important role for finding exact solutions in the mathematical physics. For these new traveling wave solutions, we give some computer simulations to illustrate our main results.
